# Supplementary material for: Defective folate metabolism causes germline epigenetic instability and distinguishes Hira as a phenotype inheritance biomarker
Source: Nat Commun. 2021 Jun 17;12:3714. doi: 10.1038/s41467-021-24036-5 (PMC8211854; doi:10.1038/s41467-021-24036-5)
Supplement: Supplementary file 1 — Supplementary Information [file 41467_2021_24036_MOESM1_ESM.pdf]

SUPPLEMENTARY INFORMATION

Defective folate metabolism causes germline epigenetic instability and distinguishes *Hira* as a phenotype inheritance biomarker

Georgina E.T. Blake[1,2,†], Xiaohui Zhao[1,2], Hong wa Yung[1,2], Graham J. Burton[1,2], Anne C. Ferguson-Smith[2,3], Russell S. Hamilton[2,3], Erica D. Watson[1,2,*]

[1]Department of Physiology, Development and Neuroscience, University of Cambridge, Cambridge UK

[2]Centre for Trophoblast Research, University of Cambridge, Cambridge UK

[3]Department of Genetics, University of Cambridge, Cambridge, UK

[†]Current address: College of Medicine and Health, University of Exeter Medical School, Exeter, UK

*Corresponding author: edw23@cam.ac.uk

Supplementary Table 1. **Common structural variants (SVs)* in all *Mtrr*$^{gt/gt}$ embryos that were absent in control C57Bl/6J embryos.**

Repeat region?	Chr	Location	Size of SV (bp)	SV type	Nearest gene	Gene location	Distance: SV and TSS (bp)	SV/ enhancer overlap?**
Yes, simple	1	80828728-80828784	56	Deletion	*Dock10*	80501073-80758527	70,201	N
Yes, simple	11	32122791-32122854	63	Deletion	*Nsg2*	32000463-32059202	63,589	N
					Il9r	32187541-32200279	77,488	N
No	15	83975384-83975519	135	Deletion	*Efcab6*	83866712-84065379	89,995	N
Tandem duplica-tion	19	36911361-37379467	468, 106	Known copy number variant in C57Bl/6J strain[1]	*Marchf5*	37207543-37222151	-	Y
					Kif11	37376403-37421859	-	Y
					Fgfbp3	36917550-36919599	-	Y
					Cpeb3	37021291-37208601	-	Y
					Btaf1	36926079-37012752	-	Y
					Ide	37268743-37334544	-	Y

*Excludes 20 Mb region surrounding *Mtrr*^{gt} locus. **Known enhancers identified via FANTOM5 software in GRCm38 mouse genome. Chr, chromosome; SV, structural variant; bp, base pairs; TSS, transcriptional start site.

Supplementary Table 2 **Characteristics of common small nucleotide polymorphisms (SNPs)* in all *Mtrr*$^{gt/gt}$ embryos that were absent in control C57Bl/6J embryos.**

Chr	Location	SNP	Context	Nearest gene	Gene location	Distance between SNP and TSS (bp)	SNP/ enhancer overlap?**
1	23478171	TC→T	Intergenic	*Ogfrl1*	23366424-23383201	94,970	N
1	130763137	A→C	Intergenic	*AA986860*	130731976-130744622	31,161	N
6	114014496	T→TCTCC CCTCCC	Intronic	*Atp2b2*	113743831-114042613	28,117	N
6	121526732	G→T	Intergenic	*Iqsec3*	121372933-121473678	53,054	N
8	98943351	A→G	Intergenic	*Gm15679*	99011886-99032740	68,535	N
8	104303432	C→G	Intronic	*Cmtm1*	104293542-104310145	6,713	N
13	58081242	C→G	Intronic	*Klhl3*	58000228-58113592	32,350	N
13	81473683	C→G	Intronic	*Adgrv1*	81095068-81633154	159,471	N
13	81473686	T→C	Intronic	*Adgrv1*	81095068-81633154	159,468	N
13	81473693	C→T	Intronic	*Adgrv1*	81095068-81633154	159,461	N
13	81473695	C→G	Intronic	*Adgrv1*	81095068-81633154	159,459	N
13	81473744	C→T	Intronic	*Adgrv1*	81095068-81633154	159,410	N
13	81473746	C→G	Intronic	*Adgrv1*	81095068-81633154	159,408	N
13	81473756	C→T	Intronic	*Adgrv1*	81095068-81633154	159,398	N
14	119813417	ACCCC→ ACCCCC	Intronic	*Hs6st3*	119138341-119869815	675,076	N
16	85907608	GC→C	Intergenic	*Adamts5*	85858157-85901125	6,483	N
16	89162602	G→A	Intergenic	*Krtap20-2*	89205861-89206394	43,259	N
18	87587984	T→C	Intergenic	*Cbln2*	86711110-86718283	876,874	N
X	68533748	TC→C	Intergenic	*Fmr1os*	68667514-68678399	144,651	N
X	71514412	C→C(A)$_{45}$	Intergenic	*Cd99l2*	71420060-71492849	21,563	N
X	169366447	TC→T	Intergenic	*Hccs*	169311530-169320374	46,073	N

*Excludes 20 Mb region surrounding the *Mtrr*^{gt} locus. **Known enhancers identified via FANTOM5 software in GRC38m mouse genome. Chr, chromosome; SNP, small nucleotide polymorphism; TSS, transcriptional start site of nearest gene.

Supplementary Table 3 **Number of sperm DMRs that validated by bisulfite pyrosequencing out of total DMRs assessed**

Male genotype*	Hypomethylated (%)	Hypermethylated (%)	Total (%)
Mtrr$^{+/+}$	0/0	3/3 (100%)	3/3 (100%)
Mtrr$^{+/gt}$	3/4 (75%)	10/20 (50%)	13/24 (54.2%)
Mtrr$^{gt/gt}$	13/13 (100%)	8/13 (61.5%)	21/26 (80.8%)
Total	16/17 (94.1%)	21/36 (58.3%)	37/53 (69.8%)

*N=8 males per experimental group including four males from MeDIP-seq analysis and four unique males. **Relative to C57Bl/6J controls. DMR, differential methylated region.

Supplementary Table 4 **Sperm DMRs identified via MeDIP-seq that were associated with known enhancer regions**

Genotype(s) in which sperm DMR identified	Chr	DMR location	Enh location	Nearest gene	Gene location (strand)	Distance: DMR and TSS (bp)	Distance: Enh and TSS (bp)
$Mtrr^{+/+}$, $Mtrr^{+/gt}$, $Mtrr^{gt/gt}$	19	37238001-37239000	37238210-37238404	*Gm25268*	37233382-37233484 (-)	4518	4727
$Mtrr^{+/gt}$, $Mtrr^{gt/gt}$	13	104660501-104661000	104660659-104660787	*Cwc27*	104631140-104817142 (-)	156143	156356
$Mtrr^{+/+}$	13	119596001-119596500	119596241-119596530	*Tmem267*	119488039-119611059 (+)	107963	108203
$Mtrr^{+/+}$	13	119597501-119598000	119597731-119598237	*Tmem267*	119488039-119611059 (+)	109463	109603
$Mtrr^{gt/gt}$	4	147275001-147275500	147275382-147275778	*Zfp988*	147305674-147333734 (+)	30175	29897
$Mtrr^{gt/gt}$	4	147738501-147739000	147738791-147739184	*Gm13157*	147753974-147809788 (-)	70789	70605
$Mtrr^{gt/gt}$	8	75083501-75084000	75083800-75084239	*Hmox1*	75093621-75100589 (+)	9622	9383
$Mtrr^{gt/gt}$	13	67797501-67798500	67797828-67797962	*4930525G20Rik*	67796594-67830985 (-)	32486	33024

*Known enhancers identified using FANTOM5 software in CRG38m mouse genome.

Supplementary Table 5 **Common sperm DMRs in *Mtrr*$^{+/+}$, *Mtrr*$^{+/gt}$ and *Mtrr*$^{gt/gt}$ males as determined by MeDIP-seq**

Chr	Start location	Stop location	Genomic location	Nearest gene	Gene location	DMR/ enhancer overlap?*
1	79722501	79723500	intronic	*Wdfy1*	79,702,262-79,776,143	N
5	14905001	14905500	intergenic	*Gm9758*	14,910,122-14,914,899	N
5	14908501	14909000	intergenic	*Gm9758*	14,910,122-14,914,899	N
5	14914501	14915000	exonic	*Gm9758*	14,910,122-14,914,899	N
5	14933501	14934000	exonic	*Speer4e*	14,933,221-14,938,429	N
5	15006001	15007000	intergenic	*Gm10354*	14,974,113-14,978,935	N
5	15031501	15032000	exonic	*Gm17019*	15,028,950-15,032,998	N
5	15040001	15040500	intergenic	*Gm17019*	15,028,950-15,032,998	N
5	15459501	15460000	intergenic	*Gm43391*	15,464,171-15,476,857	N
5	15462501	15464000	intergenic	*Gm43391*	15,464,171-15,476,857	N
5	15472501	15473500	intronic	*Gm43391*	15,464,171-15,476,857	N
5	15509001	15509500	intergenic	*Gm21847*	15,516,489-15,656,679	N
5	15522001	15522500	intronic	*Gm21847*	15,516,489-15,656,679	N
5	15527501	15528000	intronic	*Gm21847*	15,516,489-15,656,679	N
5	15528501	15529500	intronic	*Gm21847*	15,516,489-15,656,679	N
5	15596001	15597000	intronic	*Gm21847*	15,516,489-15,656,679	N
5	15601001	15601500	intronic	*Gm21847*	15,516,489-15,656,679	N
5	15630501	15631000	intronic	*Gm21847*	15,516,489-15,656,679	N
5	15632001	15632500	intronic	*Gm21847*	15,516,489-15,656,679	N
5	15637001	15638000	intronic	*Gm21847*	15,516,489-15,656,679	N
5	15643501	15644000	intronic	*Gm21847*	15,516,489-15,656,679	N
5	15670501	15671500	intergenic	*Speer4cos*	15,680,710-15,714,596	N
5	15680501	15681000	exonic	*Speer4cos*	15,680,710-15,714,596	N
5	15690501	15691000	intronic	*Speer4cos*	15,680,710-15,714,596	N
5	15713501	15714500	exonic	*Speer4cos*	15,680,710-15,714,596	N
10	122886001	122886500	intronic	*Ppm1h*	122,678,762-122,945,795	N
13	119609001	119609500	exonic	*Tmem267*	119,488,086-119,610,459	N
14	53067001	53067500	intergenic	*Trav13d-4*	53,073,004-53,073,267	N
14	53073501	53074000	intergenic	*Trav13d-4*	53,073,004-53,073,267	N
14	53624501	53625000	intergenic	*Trav12-3*	53,621,657-53,622,245	N
17	6441501	6442000	intronic	*Tmem181b-ps*	6,438,524-6,449,745	N
17	6445001	6446500	intronic	*Tmem181b-ps*	6,438,524-6,449,745	N
17	6449501	6450500	exonic	*Tmem181b-ps*	6,438,524-6,449,745	N
17	6451001	6452000	exonic	*Tmem181b-ps*	6,438,524-6,449,745	N
17	6455501	6456500	intergenic	*Tmem181b-ps*	6,438,524-6,449,745	N
17	6457001	6457500	intergenic	*Tmem181b-ps*	6,438,524-6,449,745	N
17	6463001	6464000	intergenic	*Tmem181b-ps*	6,438,524-6,449,745	N
17	6465001	6465500	intergenic	*Tmem181b-ps*	6,438,524-6,449,745	N
17	6473001	6474000	intergenic	*Tmem181b-ps*	6,438,524-6,449,745	N
17	6479001	6479500	intergenic	*Tmem181b-ps*	6,438,524-6,449,745	N
17	6502001	6502500	intergenic	*Tmem181b-ps*	6,438,524-6,449,745	N

17	6506001	6507000	intergenic	*Tmem181b-ps*	6,438,524-6,449,745	N
17	6522501	6523000	intergenic	*Tmem181b-ps*	6,438,524-6,449,745	N
17	6583501	6584000	intergenic	*Dynlt1c*	6,601,671-6,609,679	N
17	6592001	6592500	intergenic	*Dynlt1c*	6,601,671-6,609,679	N
17	58728501	58729000	intronic	*A330072L02Rik*	58,680,347-58,836,041	N
17	58730001	58731500	intronic	*A330072L02Rik*	58,680,347-58,836,041	N
19	36911001	36912000	intergenic	*Fgfbp3*	36,917,550-36,919,615	N
19	37238001	37239000	intergenic	*Gm25268*	37,207,543-37,222,151	**Y**
19	37247501	37248000	intergenic	*4931408D14Rik*	37,258,423-37,264,095	N
19	37255001	37255500	intergenic	*4931408D14Rik*	37,258,423-37,264,095	N
19	37269001	37270000	exonic	*Ide*	37,268,743-37,330,613	N
19	37277501	37278500	exonic	*Ide*	37,268,743-37,330,613	N
19	37351501	37352500	intergenic	*Ide*	37,268,743-37,330,613	N

*Enhancers identified via FANTOM5 software in GRC38m mouse genome. See also Supplementary Fig. 5.

Supplementary Table 6 **Characteristics of sperm DMRs from *Mtrr*$^{+/gt}$ males that were assessed in F1-F2 wildtype somatic tissue.**

DMR (Chromosome coordinates)	Genomic location	Repro-gramming resistant region[2,3]	Status	Nearest gene	Gene function	DMR/ enhancer overlap?*
DMR A10 (Chr16:18976001-18977000)	Intergenic	Yes	Hyper	*Hira*	Histone H3.3 chaperone	No
DMR D20 (Chr13: 104660501-104661000)	Intragenic	No	Hypo	*Cwc27*	Spliceosome-associated protein; isomerase	Yes
DMR E115 (Chr7:36770501-36772000)	Intragenic	No	Hypo	*Tshz3*	Zinc finger transcription factor	No
DMR E52 Chr17:6324501-6325500)	Intergenic	Yes	Hyper	*Dynlt1a*	Dynein light chain	No
DMR D87 (Chr17:6324501-6325500)	Intergenic	Yes	Hyper	*4930572 O03Rik*	Unknown	No
DMR D87 (Chr17:6324501-6325500)	Intergenic	Yes	Hyper	*Speer7-cos*	Spermatogenesis associated glutamate (E)-rich protein	No
DMR E112 (Chr16:33270501-33271000)	Intragenic	Yes	Hypo	*Exoc4*	Exocyst complex component	No
DMR E109 (Chr5:28168501-28169000)	Intragenic	No	Hypo	*En2*	Homeobox transcription factor	No
DMR E28 (Chr14:13512001-13512500)	Intragenic	No	Hyper	*Synpr*	Synaptoporin	No

*Known enhancers identified via FANTOM5 in GRC38m mouse genome. DMR, differentially methylated region; Hyper, hypermethylated; hypo, hypomethylated.

Supplementary Table 7 **Number of individuals assessed per CpG site in bisulfite pyrosequencing analysis for each genotype, phenotype, tissue type and generation in Fig. 3**

Generation/tissue type	F0 sperm	F0 sperm	F1 em	F1 em	F1 pl	F1 pl	F2 em	F2 em (PN)	F2 em (SA)	F2 pl	F2 pl
Genotype	C57	*+/gt*	C57	+/+	C57	+/+	C57	+/+	+/+	C57	+/+
(a)* DMR A10 [CpG sites: 5]**	3	3	3-5	4-5	6	7	4-5	4-5	3-5	6	8
(b) DMR E115 [CpG sites: 5]	8	8	5	5	7	7	5	5	5	7	8
(c) DMR D20 [CpG sites: 10]	7	6	3	3-5	7	7	3-4	4-5	3-4	7	8
(d) DMR E52 [CpG sites: 7]	8	8	5	5	6-7	7	5	5	5	3-4	8
(e) DMR E112 [CpG sites: 4]	7-8	8	5	4-5	6-7	7	3-5	5	5	4-5	7-8
(f) DMR C7 [CpG sites: 2-4]	6-8	8	4	5	6	8	5	5	5	6	8
(g) DMR E28 [CpG sites: 3]	8	8	5	3-5	6	8	5	5	5	7	8
(h) DMR E109 [CpG sites: 4]	7	8	5	5	7	7	4-5	5	4	7	5-8
(i) DMR E74 [CpG sites: 5]	7	8	5	5	7	7	5	4	5	7	8
(j) DMR D87 [CpG sites: 4]	8	8	5	5	6	7	5	5	5	7	7

See also Fig. 3.
*The Fig. 3 panel to which the data refers is shown in rounded brackets.
**The number of CpG sites assessed in the DMR is shown in square brackets.
Whole embryos (em) and placentas (pl) were assessed at embryonic day (E) 10.5.
F0, parental generation; F1, first filial generation; F2, second filial generation; C57, C57Bl/6J; +/+, *Mtrr*$^{+/+}$; *+/gt*, *Mtrr*$^{+/gt}$; PN, phenotypically normal; SA, severely affected.

Supplementary Table 8 **PCR primers used in this study.**

Gene name	Forward primer (5'→3')	Reverse primer (5'→3')	Primer conc. (nm)	Ref.
Cwc27	TGATAATGGCAGCCAGTTTTTCT	CTGTCAGGCGTAGCATGTTGT	200	-
Cts8	TCCTGTGAAGAATCAGGGCAC	GTGCTCAGTGGGACCAGTTT	200	-
Dynlt1a	GAAGACTTCCAGGCCTCCG	GGTTGACTTTGCTGTGCTGG	100	-
En2	GCTGAGTTTCAGACCAACAGGTA	GCTTGTTCTGGAACCAAATCTT	200	-
Exoc4	CACAGCCTACAGGGGCATTG	TTGGCAGCGATTTCAAGAGTC	200	-
Gapdh	CATGGCCTTCCGTGTTCCT	GCGGCACGTCAGATCCA	variable	Ref.[7]
Gas1	CCTCTGCACCACGTGTCTTA	TGGCAGTACCGAGCTTTAGG	200	-
Hira mRNA (set 1)	CTCCATCTTGTCAGGAAGTGAT	GTTCCTGGCACTCAGTAAAGAG	200	-
Hira lncRNA209 (set 2)	TATGAAAACGCCCCGCTCTT	ATTCGCGCTATTGGGCATCT	200	-
Hprt	CAGGCCAGACTTTGTTGGAT	TTGCGCTCATCTTAGGCTTT	variable	Ref.[8]
Hsd17b3	CTGAGCACTTCCGGTGAGAG	ATAAGGGGTCAGCACCTGAAT	200	-
IAP-GAG	AACCAATGCTAATTTCACCTTGGT	GCCAATCAGCAGGCGTTAGT	200	Ref.[9]
IAP-3'LTR	GCACATGCGCAGATTATTTGTT	CCACATTCGCCGTTACAAGAT	100	Ref.[9]
LINE1-5'UTR	GGCGAAAGGCAAACGTAAGA	GGAGTGCTGCGTTCTGATGA	100	Ref.[9]
LINE1-ORF2	GGAGGGACATTTCATTCTCATCA	GCTGCTCTTGTATTTGGAGCATAGA	200	Ref.[9]
March5	TTCACCAGGCTTGTCTCCA	GCATCACTGTCACTGCTCCA	150	-
Mtrr^{+}	GAGATTGGGTCCCTCTTCCAC	GCTGCGCTTCTGAATCCACAG	100	Ref.[11]
Mtrr^{gt}	GAGATTGGGTCCCTCTTCCAC	ACTTCCGGAGCGGATCTC	100	Ref.[11]
Nsun2	GGTAAACCATGACGCATCC	CCTTCCACTGTGAGACTCC	100	Ref.[10]
Ptch1	CCTCTGCTCCTTGATTGGCA	TCCCCAGTCCTGTCCTCAAA	200	-
Rbm31 (sex genotyping)	CACCTTAAGAACAAGCCAATACA	GGCTTGTCCTGAAAACATTTGG	170	Ref.[12]
SINEB1	TGAGTTCGAGGCCAGCCTGGTCTA	ACAGGGTTTCTCTGTGTAGCCCTG	100	Ref.[9]
Srd5a1	CTTGAGCCAGTTTGCGGTGTA	GCCTCCCCTGGGTATTTTGTATC	200	-
Tbpba	ACTGGAGTGCCCAGCACAGC	GCAGTTCAGCATCCAACTGCG	200	-
Tshz3	GCGCGCAGCAGCCTATGTTTC	TCAGCCATCCGGTCACTCGTC	300	-
Uqcrb	TCTCAGGTCAAAATGGCGGG	ATCATCTCGCATTAACCCCAGT	200	-

Supplementary table 9 **iPCR Tag primers used in the MeDIP-seq analysis.**

iPCR Tag primer	Primer Sequence	Barcode	Sequence Obtained
iPCRtag1	CAAGCAGAAGACGGCATACG**AGAT**AACGTGAT**GAGA**T CGGTCTCGGCATTCCTGCTGAACCGCTCTTCCGATC	AACGTGAT	ATCACGTT
iPCRtag2	CAAGCAGAAGACGGCATACGAGATAAACATCGGAGAT CGGTCTCGGCATTCCTGCTGAACCGCTCTTCCGATC	AAACATCG	CGATGTTT
iPCRtag3	CAAGCAGAAGACGGCATACGAGATATGCCTAAGAGAT CGGTCTCGGCATTCCTGCTGAACCGCTCTTCCGATC	ATGCCTAA	TTAGGCAT
iPCRtag4	CAAGCAGAAGACGGCATACGAGATAGTGGTCAGAGAT CGGTCTCGGCATTCCTGCTGAACCGCTCTTCCGATC	AGTGGTCA	TGACCACT
iPCRtag5	CAAGCAGAAGACGGCATACGAGATACCACTGTGAGAT CGGTCTCGGCATTCCTGCTGAACCGCTCTTCCGATC	ACCACTGT	ACAGTGGT
iPCRtag6	CAAGCAGAAGACGGCATACGAGATACATTGGCGAGAT CGGTCTCGGCATTCCTGCTGAACCGCTCTTCCGATC	ACATTGGC	GCCAATGT
iPCRtag7	CAAGCAGAAGACGGCATACGAGATCAGATCTGGAGAT CGGTCTCGGCATTCCTGCTGAACCGCTCTTCCGATC	CAGATCTG	CAGATCTG
iPCRtag8	CAAGCAGAAGACGGCATACGAGATCATCAAGTGAGAT CGGTCTCGGCATTCCTGCTGAACCGCTCTTCCGATC	CATCAAGT	ACTTGATG

Supplementary table 10 **Source and accession numbers of processed ChIP-seq and ATAC-seq wig/bigwig files**

ID	Experiment	Sample	Target	GEO Accession	Source	Ref.	Data
1	ChIP-seq	ESC	H3K27ac	GSM1000099	ENCODE/PMID: 30305613	Ref.[4]	raw/ processed*
2	ChIP-seq	ESC	H3K4me3	GSM769008	ENCODE/PMID: 30305613	Ref.[4]	raw/ processed*
3	ChIP-seq	ESC	H3K9me3	GSM1000147	ENCODE/PMID: 30305613	Ref.[4]	raw/ processed*
4	ChIP-seq	ESC	H3K4me1	GSM769009	ENCODE/PMID: 30305613	Ref.[4]	raw/ processed*
5	ChIP-seq	ESC	H3K27me3	GSM1000089	ENCODE/PMID: 30305613	Ref.[4]	raw/ processed*
6	ChIP-seq	ESC	CTCF	GSM918748	ENCODE/PMID: 30305613	Ref.[4]	raw/ processed*
8	ChIP-seq	TSC	H3K27ac	GSM1035380, GSM1035381	PMID: 23396136	Ref.[4]	raw/ processed*
9	ChIP-seq	TSC	H3K4me3	GSM1035382	PMID: 23396136	Ref.[4]	raw/ processed*
10	ChIP-seq	TSC	H3K9me3	GSM1035383, GSM1035384	PMID: 23396136	Ref.[4]	raw/ processed*
11	ChIP-seq	TSC	H3K4me1	GSM1035385	PMID: 23396136	Ref.[4]	raw/ processed*
12	ChIP-seq	TSC	H3K27me3	GSM1035386, GSM1035387	PMID: 23396136	Ref.[4]	raw/ processed*
13	ChIP-seq	TSC	CTCF	GSM967658	PMID: 23178118	Ref.[4]	raw/ processed*
15	ChIP-seq	Sperm	H3K27ac	GSM2088387, GSM2401435	PMID: 28178516	Ref.[5]	processed
16	ChIP-seq	Sperm	H3K4me3	GSM2088391, GSM2401439	PMID: 28178516	Ref.[5]	processed
17	ChIP-seq	Sperm	H3K9me3	GSM2088388, GSM2401436	PMID: 28178516	Ref.[5]	processed
18	ChIP-seq	Sperm	H3K4me1	GSM2088390, GSM2401438	PMID: 28178516	Ref.[5]	processed
19	ChIP-seq	Sperm	H3K27me3	GSM2088386, GSM2401434	PMID: 28178516	Ref.[5]	processed
20	ChIP-seq	Sperm	CTCF	GSM2088382, GSM2088383, GSM2088384	PMID: 28178516	Ref.[5]	processed
21	ChIP-seq	Sperm	H3	GSM2088392	PMID: 28178516	Ref.[5]	processed
22	ChIP-seq	Sperm	PRM1	GSM2088400, GSM2401441	PMID: 28178516	Ref.[5]	processed
23	ATAC-seq	Sperm	THSS	GSM2088376, GSM2088377, GSM2088378	PMID: 28178516	Ref.[5]	processed
24	ATAC-seq	ExE	THSS	GSM2229962, GSM2229963	PMID: 28959968	Ref.[6]	processed
25	ATAC-seq	Epi	THSS	GSM2229960, GSM2229961	PMID: 28959968	Ref.[6]	processed

*We used the processed file from the author directly. ExE, extraembryonic ectoderm (E6.5); Epi, epiblast (E6.5). All files accessible on GitHub: https://github.com/CTR-BFX/Blake_Watson

Supplementary Table 11 **Bisulfite pyrosequencing primers used in this study.**

DMR name	Coordinates	Forward primer* (5'→3')	Reverse primer (5'→3')	Sequencing primer** (5'→3')
11	Chr1: 65,104,501-65,105,000	[biotin]-ATAGATAGTGAAAGGATGGAGAGTAAGAA	CTCCAACCACTAAAAACTTAACTCA	CCACTAAAAACTTAACTCAATC
17	Chr2: 144,308,501-144,309,000	AGTTTTTTTGGGTGTGAGAATTAT	[biotin]-ATCCAAAACCACTAAAAAACACAAC	TGGGTGTGAGAATTATT
29	Chr19: 36,911,501-36,912,000	AGGGGAGAATTTTGAATGTGAT	[biotin]-ACCCCACAACTTTCTTATAACTT	GGTTATAGGGGGGAT
60	Chr10: 122,886,001-122,886,500	[biotin]-AGATGTAAAAGAAAGGAAGGTAGT	CAATCCCCCATTCAATACAAAAA	ACAAAAATACCCTCCC
177	Chr19: 37,247,501-37,248,000	[biotin]-AATTTAAGTGAAGAATTTTGGTTTTATG	AACCCTAAATATTCTCCTTTACTCAAC	ATTCTCCTTTACTCAACT
181	Chr19: 37,280,001-37,280,500	AGGAAGTATTTGAGATGTTTAGAGTT	[biotin]-CCACTACAATATATCCTTAACCCTACT	TTATAGGATTATAGGATGTGT
185	Chr3: 122,504,501-122,505,000	[biotin]-TGTTTTATTTGTAGTTGGAGAAGTAAG	ACCTCTCAAAAATCCTATCCATAATAT	CACCCCCTCACTAATCCTCTA
189	Chr4: 156,135,001-156,136,000	TGGGAGTTAGTTATTGGTTTAGTTTGAGG	[biotin]-AACCCAATACTAATCCACCTTTACA	TGAGGTTGGTATAGGAA
220	Chr6: 136,907,001-136,907,500	AGTTATGTGTAGGGTATTAATTTAATGT	[biotin]-TCTCCCCATACCTAACTTCACACT	AGATGTATGTTTTTGAAGTTTA
269	Chr6: 33,270,501-33,271,000	[biotin]-TAGGAAATAAAAATGTGAGGGGTAAT	ACTACTACTCTATCCCTTTTATAACA	ACTCTATCCCTTTTATAACAA
274	Chr7: 75,821,501-75,822,000	[biotin]-GGATGTAGATGAATTTGGAAATTTTAGA	ACTAAAACTCCACTATTCCTTTCCACAATT	CTATTCCTTTCCACAATTAC
278	Chr8: 116,801,001-116,801,500	GTTTGAATTTTTTGAGGGTTATTTAAGGTT	[biotin]-TCCCCCTTTTAAAACTATCTCTTCT	TTTGAGTTTAGTTTTTTATATTGTG
279	Chr8: 119,794,501-119,795,000	GTTTTTTTAGTAGAGTTGGGAGTTT	[biotin]-AAACAAACTAAACCTAAACAAATATAACT	GGGAGTTTTGTTTTTTTTAGAT
280	Chr10: 4,354,501-4,355,000	AGAAGGGGTTATAGGAAGTATTTTAGG	[biotin]-CCCAAAAAATAATCCCTTCCCTTTTC	GGGTTATAGGAAGTATTTTAGGA
281	Chr7: 75,821,501-75,822,000	AGGATGGGGGAATAAAATGATG	[biotin]-ACCCAAACCTATATAATAATAACTTTCC	GGGGAATAAAATGATGTG
282	Chr10: 82,975,501-82,976,500	GAATTGTTTAGGGAGGATTTTTTTATAGT	[biotin]-CCAACATTCTACAAAATCTACTACTCC	TGTTGTGTAGATAGATATTTTGAT
289	Chr11: 44,696,501-44,697,000	GGTTGTGGGTTGTTATTGTAGTTAAG	[biotin]-ATCAAATTTTCTCTTTCCCCATTC	GTTATTGTAGTTAAGGGGT
A10	Chr16: 18,975,501-18,977,000	TGTTATAGGTTGGAGGGTATGAGTT	[biotin]-ATCCAATCCTTCTCAACAATCTCTCTC	AGGAGTAGAGAGTTTTAG
B19	Chr19: 37,252,001-37,252,500	[biotin]-AATTGGAGAATAGGATTTTTAGTGTTTATA	AACAATAATAAATACTTAAACTACA	TCCTAAATCTCCCTATAACACCATCAAT
B21	Chr19: 37,260,501-37,261,000	AGTATAAGTGATGGAGTTTTAGATAAAT	[biotin]-AATCTTAAACCAATACAAATCCATACT	TGATGGAGTTTTAGATAAATTAG

C7	Chr19: 37,238,001-37,239,000	GGTAGTTTTGGATAT GTAAGAGTTG	[biotin]-CATATCCCCTCTCTCT TTATCTTTTTA	GGTTTATAGAGTTGAGA ATTGTG
D20	Chr13: 104,660,501-104,661,000	GAAATGTTTGAGAGA ATGGGTTTTAG	[biotin]-ATACAATAACCTTAAC CACTCATAAC	AAATTTTAGTTTAAGGT TTTATATA
D41	Chr19: 37,247,501-37,248,000	[biotin]-AAGTGAAGAATT TTGGTTTTATGTAGA AT	AACCCTAAATATTCTC CTTTACTCAACT	CTCCTTTACTCAACTCT
D87	Chr5: 15,671,001-15,671,500	GGGTGTTGTTTTAGA GGTTTTGTATTTTTG	[biotin]-ATCACCTCATC CTACTAAATACTATAA C	AGATTAATTTTTTAAGT TTTGTTG
E28	Chr14: 13,512,001-13,512,500	TAGGGAGATTGATTG TGTTTTATTAGAT	[biotin]-ACTACCCTCTTCCTCT CTTACTCCTAAAT	AGATTGATTGTGTTTTA TTAGATT
E50	Chr15: 78,741,501-78,742,000	AGGTAGATTAGGTTG AATATTTATAGAGA	[biotin]-AAAAACAAAATCTATA AAACCCTAAATCC	GGGTTGAAATTAAAGAT AGG
E52	Chr17: 6,324,501-6,325,500	AGTTTTGGAGTTTAG GTTAGGT	[biotin]-ATAACCATATCAATAA AACCTTCTTAACA	AGTTTAGGTTAGGTTAA AG
E66	Chr17: 6,562,501-6,563,000	GGAGAGGGTTGTTTT TTTTGGGATAAGT	[biotin]-ATTTACCCTTCTCCCC ACTACCA	GTGTTTTTGTTTTTAGT TAGTTATT
E74	Chr18: 47,505,501-47,506,000	GTAAGTTGGAGGTAG ATATAGTTAGAAGT	[biotin]-ACTCCATTTACAACTC TTCACCATACT	ATAAAAGTTTTTAAATT TATTTGGG
E81	Chr2: 135,170,501-135,171,000	TGTAAGGTTTAAGAT AGGAGATGTTATT	[biotin]-TACAAACCCATATAAC TACAAAACTCAT	TTTAGTTGGTGAGATTG A
E109	Chr5: 28,168,501-28,169,000	TAGAAGTTTTATTGG TTAGGTGTAAGTTA	[biotin]-CAAAAAAATCTTTAAA CACCAAAAAACATC	ATGGATGTGAAGAGG
E112	Chr6: 33,270,501-33,271,000	[biotin]-TAGGAAATAAAAATG TGAGGGGTAAT	ACTACTACTCTATCCC TTTTATAACA	ACTCTATCCCTTTTATA ACAAT
E114	Chr7: 16,633,501-16,634,000	GTTTTTGGGTTTTAG TAATAGTGTATGG	[biotin]-AACACATATAACTTCC CACACATC	GTTTTTAGAGAGTAGTT TAGG
E115	Chr7: 36,770,501-36,772,000	AGTTTGATATTGAAG GGGTTATTTTGGA	[biotin]-AACATAATATACTTCC CTTCTAAAATCT	GGTTATTTTGGAAGAGG T

Primer concentration: *PCR primers, 250 nM; **Sequencing primers, 417 nM

SUPPLEMENTARY FIGURES AND LEGENDS

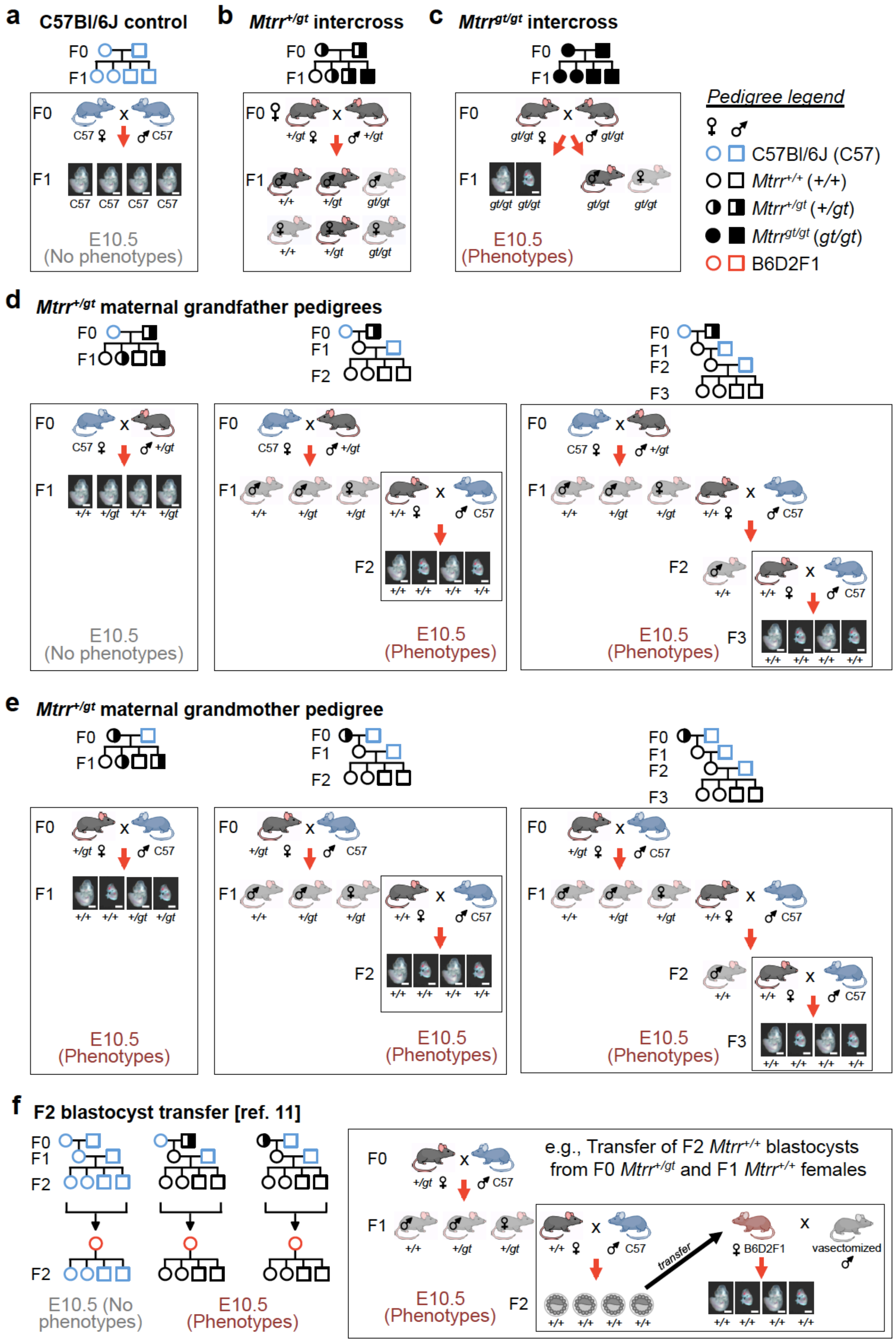

Supplementary Figure 1 **Genetic pedigrees and breeding schemes used in this study and a published study[11]. a** C57Bl/6J (C57) intercross that generated the controls used in all analyses. **b** *Mtrr*$^{+/gt}$ (*+/gt*) intercross that generated *Mtrr*$^{+/+}$ (+/+) and *Mtrr*$^{+/gt}$ male mice used in the sperm analysis (see also Figs. 2, 3, 5b-c and Supplementary Figs. 4-6) and to generate F0 *Mtrr*$^{+/gt}$ mice (see also Figs. 3-4, 6 and Supplementary Fig. 11). **c** *Mtrr*$^{gt/gt}$ (*gt/gt*) intercross used to generate F1 *Mtrr*$^{gt/gt}$ embryos and placentas at embryonic day (E) 10.5 for whole genome sequencing (see also Fig. 1, Supplementary Fig. 3) and for methylation and transcription analyses (see also Supplementary Figs. 8, 11), and to generate *Mtrr*$^{gt/gt}$ males for the sperm analysis (see also Figs. 2, 3, 5b-c and Supplementary Figs. 4-6). **d** *Mtrr*$^{+/gt}$ maternal grandfather pedigree used to generate F1, F2 and F3 *Mtrr*$^{+/+}$ embryos and placentas for molecular analyses (see also Figs. 3-4, 6 and Supplementary Fig. 11). **e** *Mtrr*$^{+/gt}$ maternal grandmother pedigree used to generate F1, F2, and F3 *Mtrr*$^{+/+}$ embryos and placentas for molecular analyses (see also Fig. 6 and Supplementary Fig. 11). **f** Blastocyst transfer experiment performed in a previous study[11] to demonstrate germline inheritance of a yet-to-be determined epigenetic factor that causes increased risk of congenital malformations at E10.5 in the *Mtrr*^{gt} mouse line over multiple generations. Three pedigrees were involved in this experiment including the C57Bl/6J (C57) control pedigree (left-hand pedigree), *Mtrr*$^{+/gt}$ maternal grandfather pedigree (centre pedigree) and *Mtrr*$^{+/gt}$ maternal grandmother pedigree (right-hand pedigree). The breeding scheme and blastocyst transfer protocol is shown in the boxed region to the right using the *Mtrr*$^{+/gt}$ maternal grandmother pedigree as an example. The F2 wildtype blastocysts derived from an F0 *Mtrr*$^{+/gt}$ female (mated to C57Bl/6J male) and F1 *Mtrr*$^{+/+}$ female (mated to C57Bl/6J male) were transferred into the control uteri of [C57Bl/6J x DBA/2] F1 hybrid (B6D2F1) pseudopregnant females (mated with vasectomized C57Bl/6 males). This experiment showed that congenital malformations (and not growth phenotypes) formed at E10.5 and thus were independent of the F1 uterine environment. See also ref. 11 for more information. Scale bar: 500 μm. F0, parental generation; F1, first filial generation; F2, second filial generation; F3, third filial generation. Pedigree legend: circle, female; square, male; blue outline, C57Bl/6J mouse line; black outline, *Mtrr*^{gt} mouse line; red outline, B6D2F1 mouse line; white fill, *Mtrr*$^{+/+}$; half black-half white fill, *Mtrr*$^{+/gt}$; black fill, *Mtrr*$^{gt/gt}$.

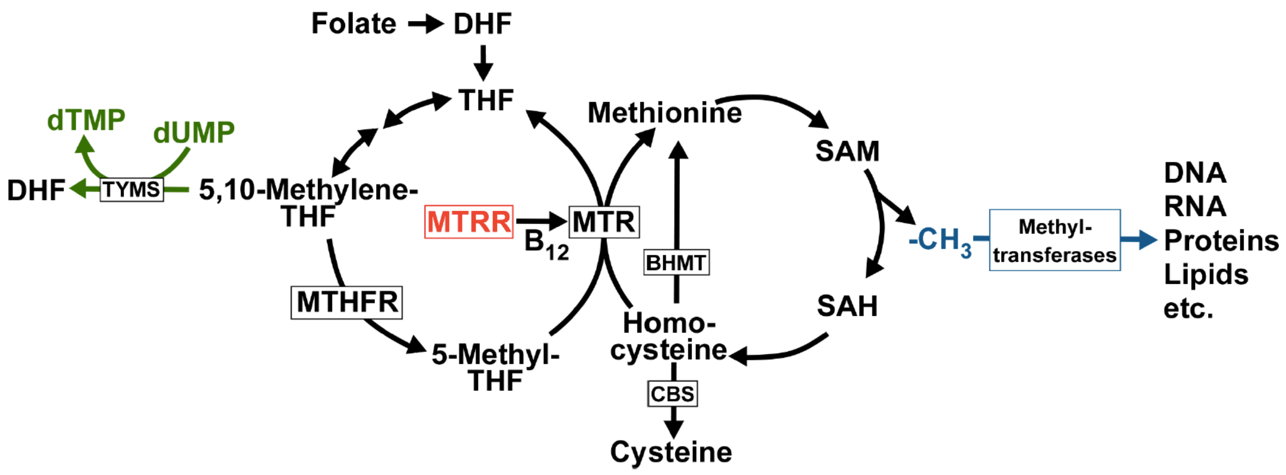

Supplementary Figure 2 **Simplified schematic drawing of one-carbon metabolism.** Folate metabolism is involved in thymidine synthesis and the transmission of one-carbon methyl groups for cellular methylation reactions. Methionine synthase reductase (MTRR; red) is a key enzyme at the intersection between folate and methionine pathways that is required to activate methionine synthase (MTR) through the reductive methylation of its vitamin B_{12} cofactor. BHMT, betaine-homocysteine S-methyltransferase; CBS, cystathionine beta-synthase; $-CH_3$, one-carbon methyl group; DHF, dihydrofolate; dTMP, deoxythymidine monophosphate; dUMP, deoxyuradine monophosphate; MTHFR, methylenetetrahydrofolate reductase; SAH, S-adenosyl-homocysteine; SAM, S-adenosyl-methionine; THF, tetrahydrofolate; TYMS, thymidylate synthase.

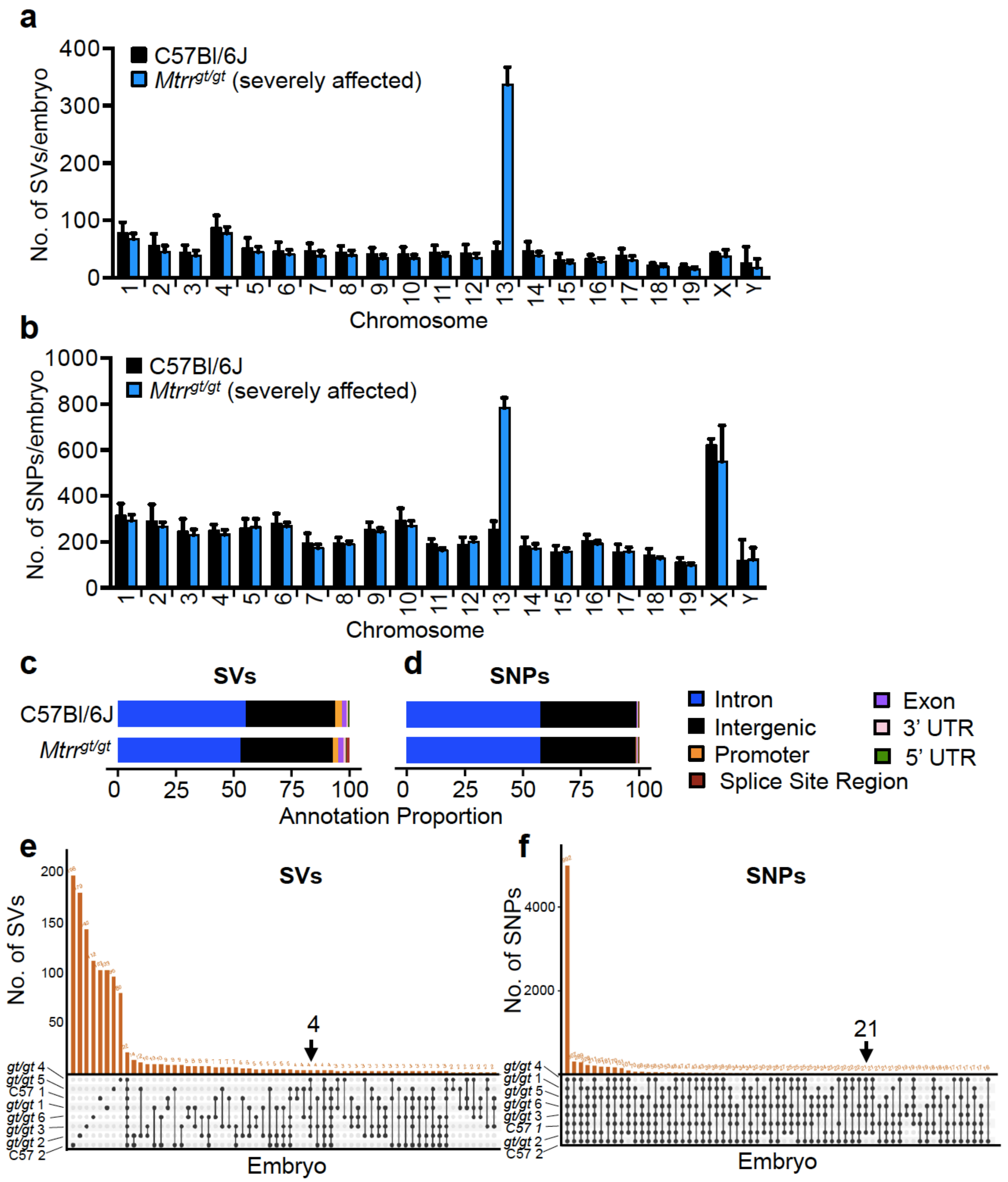

Supplementary Figure 3 **Frequency and location of genetic variants in C57Bl/6J and *Mtrr*$^{gt/gt}$ embryos. a, b** Whole genome sequencing data showing the average frequency of **a** structural variants (SVs) and **b** single nucleotide polymorphisms (SNPs) for each chromosome. Phenotypically normal C57Bl/6J embryos (N=2, black bars) and severely affected *Mtrr*$^{gt/gt}$ embryos (N=6, blue bars) were assessed. Data is presented as mean ± standard deviation. Note, the gene-trap (gt) insertion in the *Mtrr* locus was on Chr13. **c, d** Genomic location of **c** SVs and **d** SNPs in C57Bl/6J and *Mtrr*$^{gt/gt}$ embryos after masking the region surrounding the gene-trap insertion site. **e,**

f UpsetR plots showing intersections of **e** SVs or **f** SNPs between individual C57Bl/6J (C57) and *Mtrr*$^{gt/gt}$ (*gt/gt*) embryos. Arrows indicate the variants present in all *Mtrr*$^{gt/gt}$ embryos and not C57Bl/6J embryos. See also Supplementary Tables 1 and 2. UTR, untranslated region.

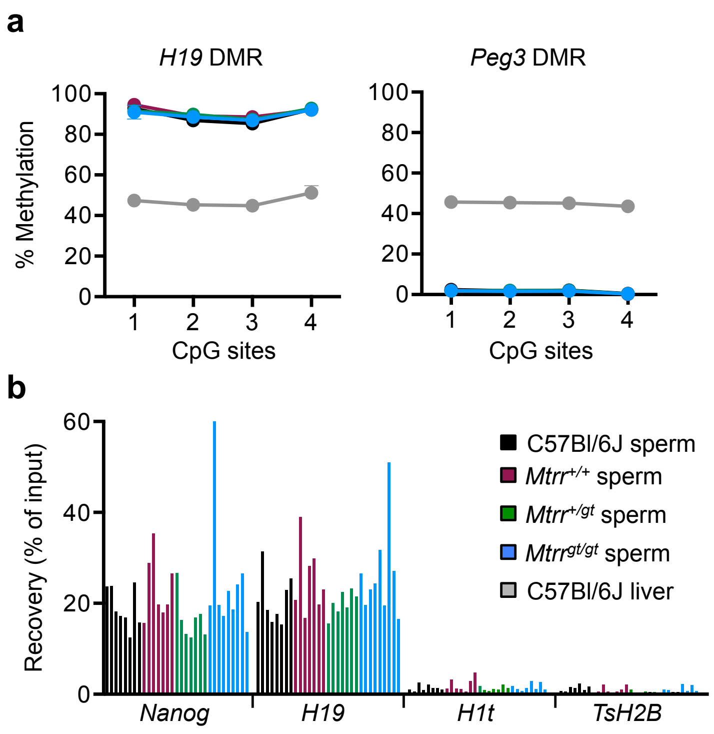

Supplementary Figure 4 **Confirmation of spermatozoa purity and validation of immunoprecipitation. a** Bisulphite pyrosequencing of imprinting control regions in DNA from spermatozoa collected from cauda epididymides to determine sperm purity. Percentage methylation at a each CpG site in the maternally imprinted *Peg3* differentially methylated region (DMR) and paternally imprinted *H19* DMR were determined in sperm samples isolated from C57Bl/6J (black circles), wildtype (*Mtrr*$^{+/+}$; purple circles), *Mtrr*$^{+/gt}$ (green circles) and *Mtrr*$^{gt/gt}$ (blue circles) mice. C57Bl/6J liver (grey dots) was assessed as a control. Data is represented as mean ± standard deviation for each CpG site. N=8 males/tissue type/genotype. **b** Percentage recovery of DNA input after methylated DNA immunoprecipitation (MeDIP) experiment as determined using quantitative reverse transcription PCR (RT-qPCR) to amplify known methylated (*Nanog* and *H19*) and unmethylated (*H1t* and *TsH2B*) regions. MeDIP samples from sperm of C57Bl/6J (black bars), *Mtrr*$^{+/+}$ (purple bars), *Mtrr*$^{+/gt}$ (green bars), and *Mtrr*$^{gt/gt}$ (blue bars) males are shown. Each bar indicates one individual (N=8 males/tissue type/genotype). Source data are provided as a Source Data file.

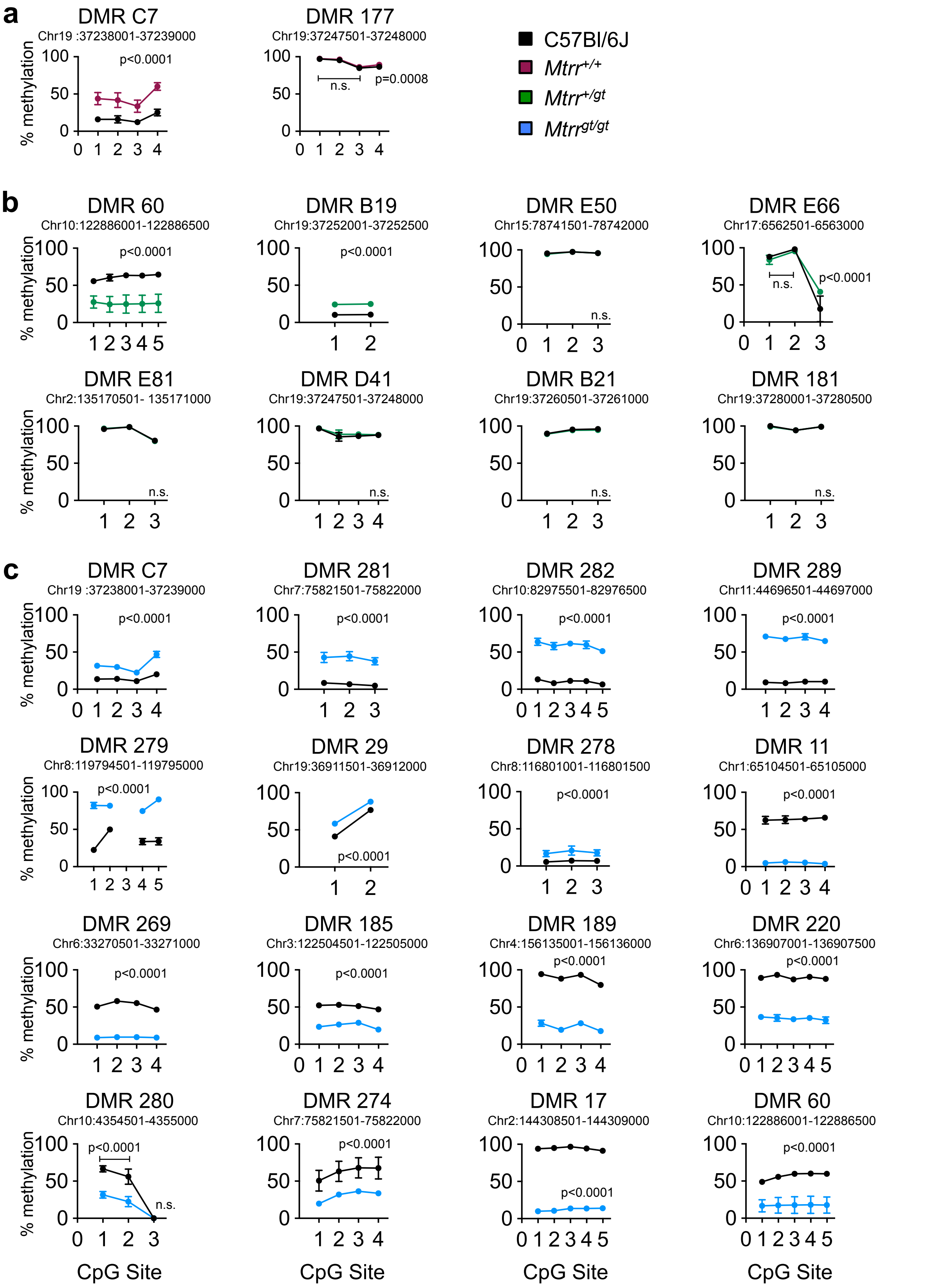

Supplementary Figure 5 **Validation of a panel of differentially methylated regions (DMRs) identified in sperm of *Mtrr*$^{+/+}$, *Mtrr*$^{+/gt}$ and *Mtrr*$^{gt/gt}$ males relative to C57Bl/6J controls. a-c** Validation of DMRs identified in a methylated DNA immunoprecipitation assay followed by DNA sequencing (MeDIP-seq) experiment of sperm from wildtype (*Mtrr*$^{+/+}$; purple circles), *Mtrr*$^{+/gt}$ (green

circles) and *Mtrr*$^{gt/gt}$ (blue circles) males relative to C57Bl/6J control sperm (black circles). The average percentage methylation at individual CpG sites was determined by bisulfite pyrosequencing. Data is plotted as mean ± standard deviation at each CpG site. N=8 males per genotype (four samples from MeDIP-seq analysis plus four unique samples). The ID and chromosome (Chr) coordinates are given for each DMR. Two-way ANOVA, with Sidak's multiple comparisons test, performed on mean methylation per CpG site per genotype group. p values indicated are for all CpG site comparisons within the DMR unless otherwise indicated. n.s., not significant. Source data are provided as a Source Data file.

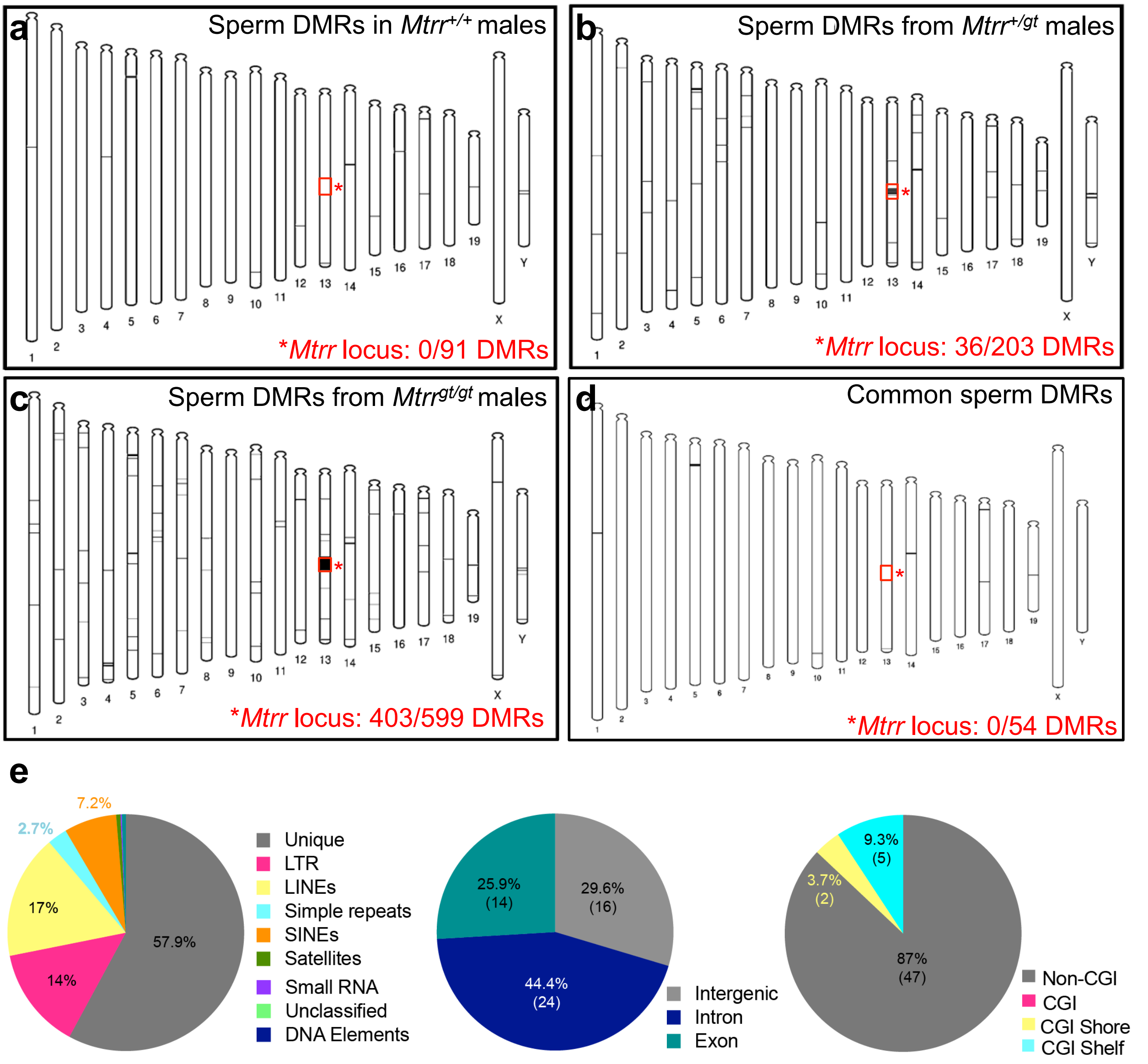

Supplementary Figure 6 **Chromosomal distribution of differentially methylated regions (DMRs) in sperm of *Mtrr*$^{+/+}$, *Mtrr*$^{+/gt}$, and *Mtrr*$^{gt/gt}$ males relative to C57Bl/6J control males. a-d** Phenograms showing chromosomal location of sperm DMRs (black lines/rectangles) identified via MeDIP-seq analysis in **a** wildtype (*Mtrr*$^{+/+}$), **b** *Mtrr*$^{+/gt}$, and **c** *Mtrr*$^{gt/gt}$ males compared to C57Bl/6J sperm. **d** A phenogram depicting the chromosome location of the 54 common sperm DMRs between *Mtrr* genotypes when compared to C57Bl/6J sperm. Red box in **a-d** indicates region on chromosome 13 surrounding the *Mtrr* locus identified in Fig. 1a. **e** Relative genomic distribution of methylated regions among the 54 common sperm DMRs between *Mtrr*$^{+/+}$, *Mtrr*$^{+/gt}$ and *Mtrr*$^{gt/gt}$ males (compared to C57Bl/6 males) with respect to unique sequences and repetitive elements, coding and non-coding regions, and CpG islands (CGIs), shores and shelves (see also Fig. 2e-f). N=8

males per genotype. LTR, long terminal repeat; LINEs, long interspersed nuclear elements; SINEs, short interspersed nuclear elements.

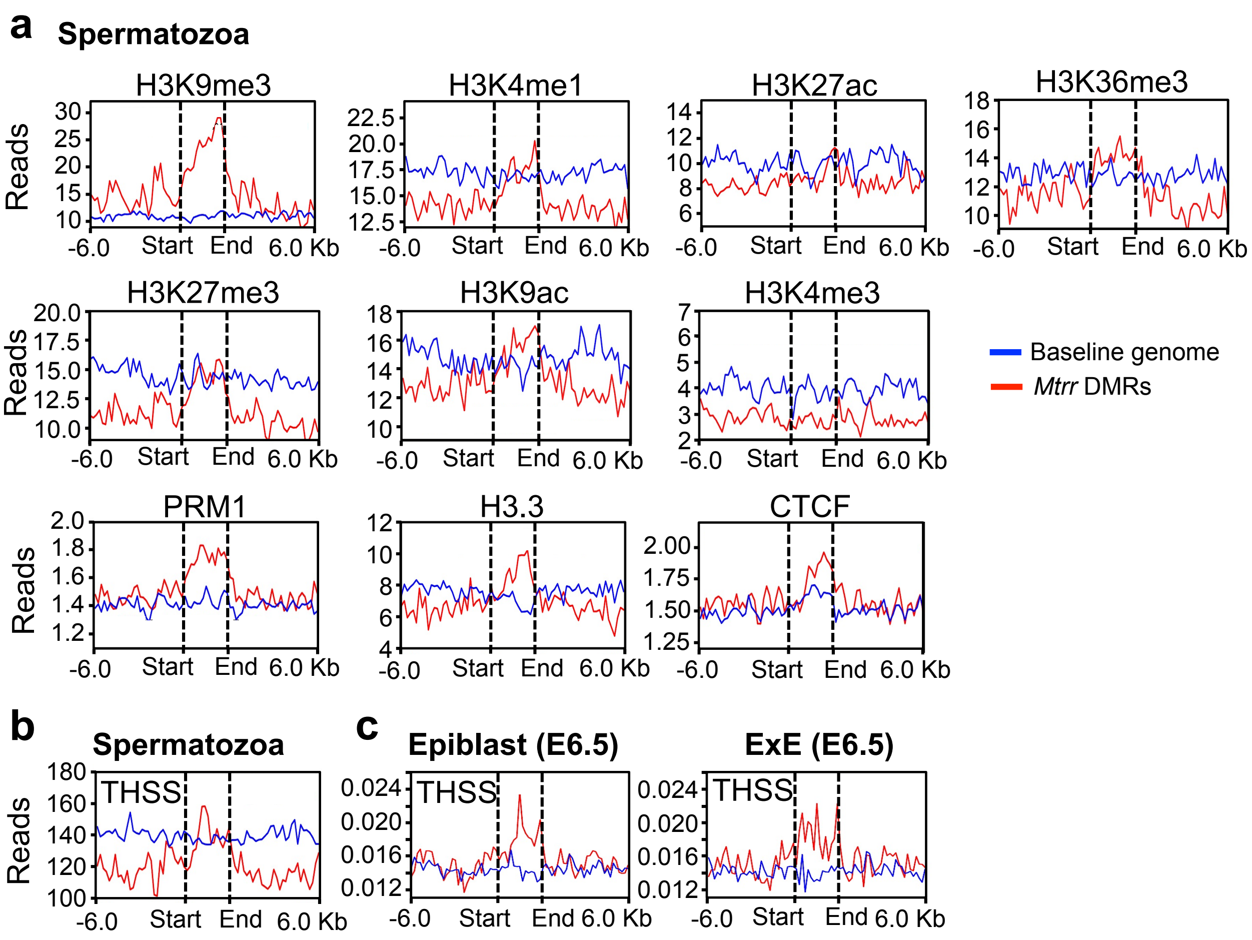

Supplementary Fig. 7 **General epigenetic signature of genomic regions identified as sperm DMRs in *Mtrr* males. a** Using published ChIP-seq (chromosome immunoprecipitation followed by DNA sequencing) data sets in wildtype CD1 mouse spermatozoa[5], mean enrichment of selected histone modifications and DNA binding proteins was determined in the genomic regions identified as differentially methylated in sperm. Differentially methylated regions (DMRs) of all *Mtrr* genotypes, excluding those within the 20 Mb (megabase) region surrounding the *Mtrr* locus, were combined in this analysis (N= 379 DMRs; red line) and compared to the baseline genome (blue line; see Methods). **b, c** Using published ATAC-seq (assay for transposase-accessible chromatin with high throughput sequencing) data sets in **b** wildtype CD1 mouse spermatozoa[5] and **c** wildtype [C57Bl/6J x DBA/2] F1 hybrid (B6D2F1) mouse epiblast and extraembryonic ectoderm (ExE) at embryonic day (E) 6.5 [ref.[6]], mean enrichment of Tn5 transposase sensitive site (THSS) was determined in sperm DMRs of all *Mtrr* genotypes combined (N= 379 DMRs; red line) compared to the baseline genome (blue line). DMRs in the region surrounding the *Mtrr* gene-trap insertion site were masked in both analyses. Dotted lines indicate the start and end of the DMR. Six kilobases

(Kb) of DNA surrounding the DMR was also considered. PRM1, protamine 1; H3.3, H3 histone, family 3; CTCF, CCCTC-binding factor.

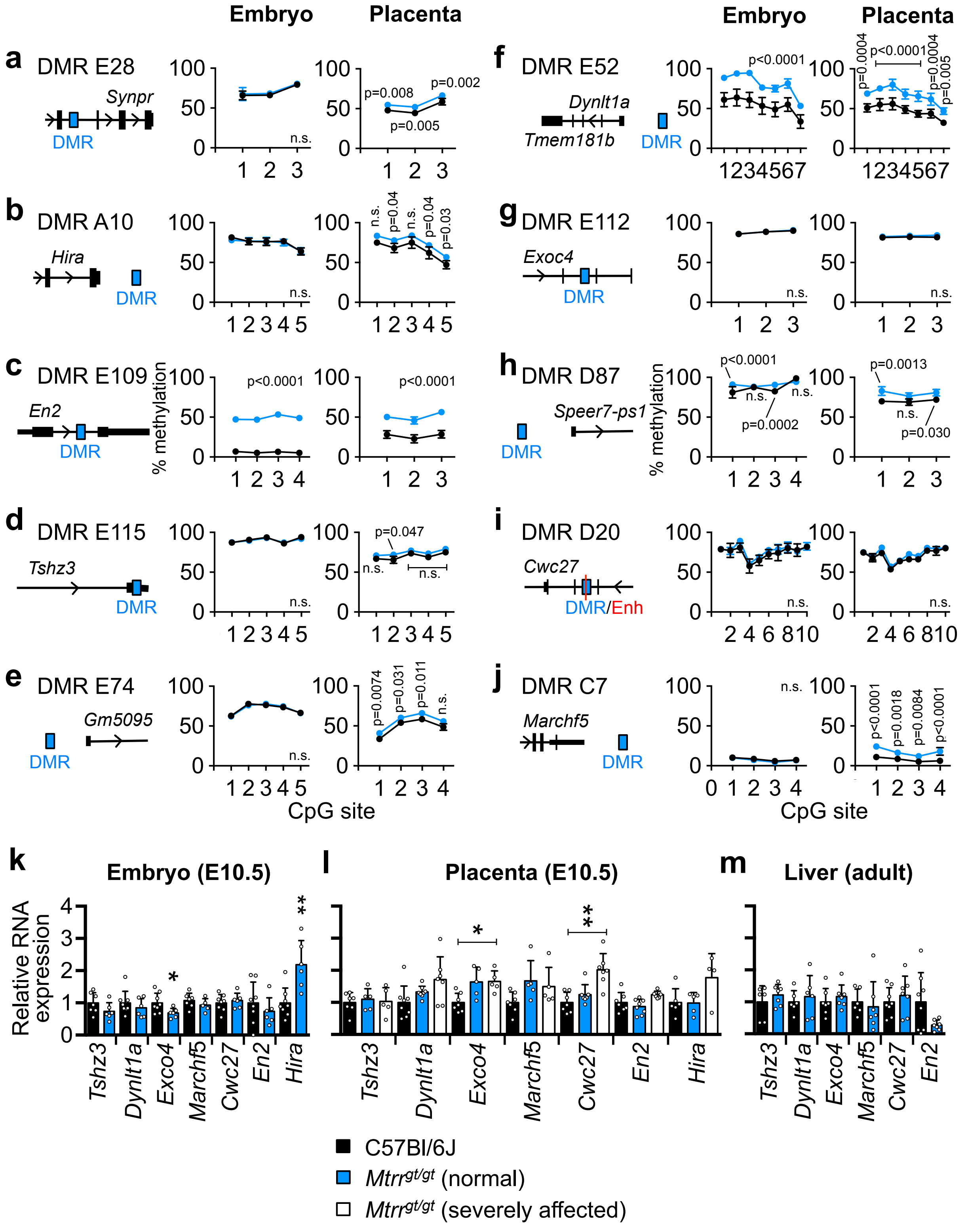

Supplementary Figure 8 **Analysis of DNA methylation and gene expression at sperm differentially methylated regions (DMRs) in *Mtrr*$^{gt/gt}$ tissue. a-j** Schematic drawings of DMRs (blue rectangles) identified in sperm from *Mtrr*$^{+/gt}$ males in relation to the closest gene alongside bisulfite pyrosequencing analysis of CpG methylation at these DMRs in C57Bl/6J control (black circles) and *Mtrr*$^{gt/gt}$ (blue circles) phenotypically normal embryos and placentas at embryonic day

(E) 10.5. The average percentage of methylation at individual CpG sites within the corresponding DMR is shown (mean ± standard deviation (sd) for each CpG site). Enhancer (Enh) overlap is shown in red. For each CpG site/DMR, C57Bl/6J: N=6 embryos, N=4 placentas. *Mtrr*$^{gt/gt}$: N=8 embryos, N=3-4 placentas. Two-way ANOVA, with Sidak's multiple comparisons test, performed on mean methylation per CpG site per genotype group. p values are indicated for all CpG sites assessed unless noted for individual CpG site. n.s., not significant. **k-m** Quantitative reverse transcription PCR (RT-qPCR) analysis of mRNA expression of some of the genes proximal to or overlapping with sperm DMRs (shown in a-f). Gene expression was assessed in C57Bl/6J control (black bars) and *Mtrr*$^{gt/gt}$ (blue and white bars) tissue including **k** embryos and **l** placentas at E10.5, and **m** *Mtrr*$^{gt/gt}$ adult livers. Placentas from phenotypically normal (blue bars) and severely affected (white bars) *Mtrr*$^{gt/gt}$ embryos were assessed. Embryos: C57Bl/6J, N=7-8 embryos; *Mtrr*$^{gt/gt}$, N=5-6 embryos. Placentas: C57Bl/6J, N=6-8 placentas; *Mtrr*$^{gt/gt}$, N=5-8 placentas from phenotypically normal conceptuses, N=4-7 placentas from severely affected conceptuses. Liver: C57Bl/6J, N=6-8 livers; *Mtrr*$^{gt/gt}$, N=6-7 livers. RT-qPCR data is plotted as mean ± sd, and relative to C57Bl/6J levels (normalized to 1). Independent two-tailed t tests or one-way ANOVA were performed. k, *p=0.0384, **p=0.0021; l, *p=0.01, **p=0.001. Source data are provided as a Source Data file.

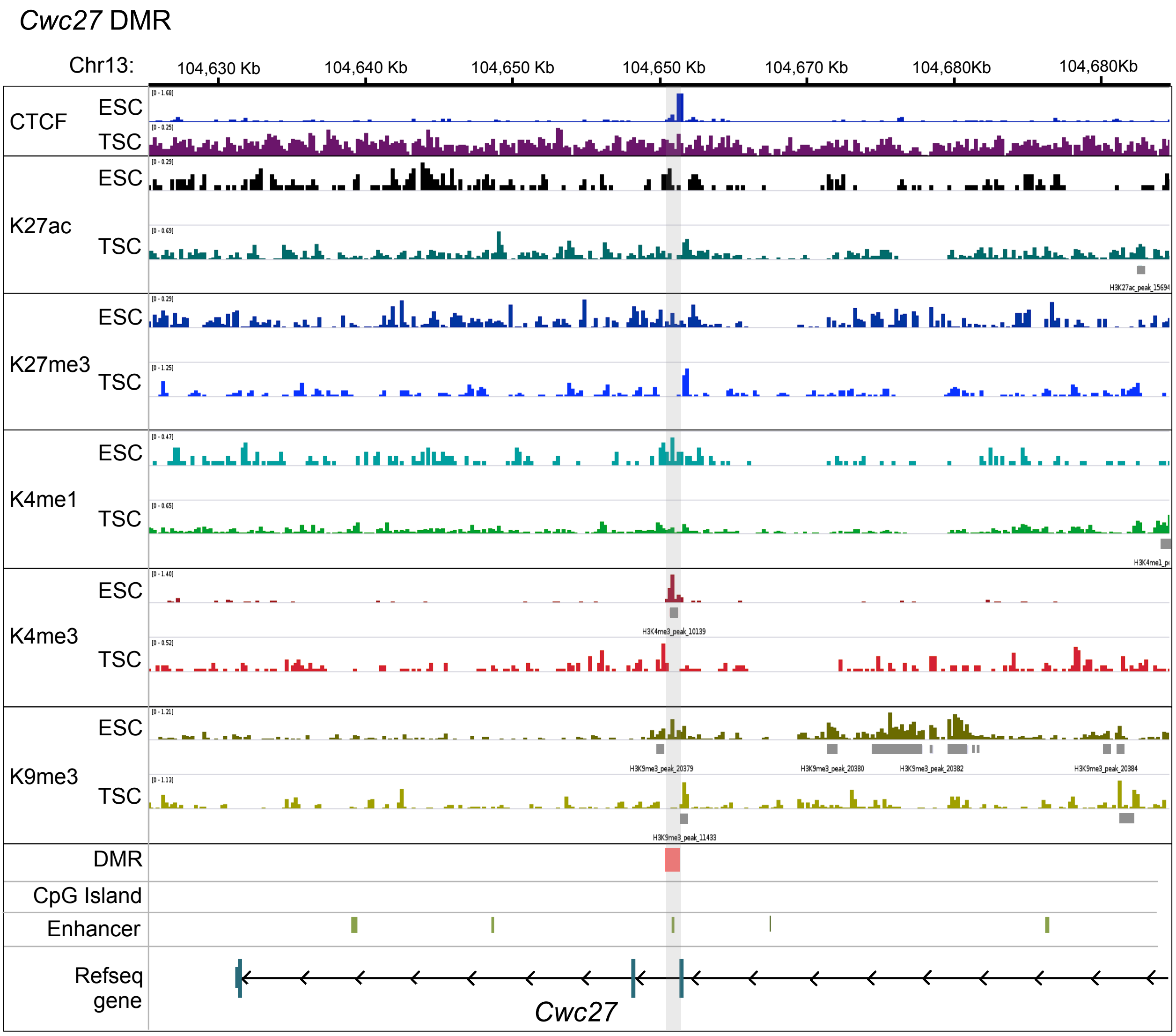

Supplementary Figure 9 **Epigenetic signature of the intragenic *Cwc27* DMR in wildtype mouse ESCs and TSCs.** Enrichment of DNA binding proteins (CCCTC-binding factor, CTCF) and histone modifications (H3K27ac, H3K27me3, H3K4me1, H3K4me3, H3K9me3) in the *Cwc27* (CWC27 spliceosome-associated protein) locus on chromosome (Chr) 13 (~37,000 kb downstream of *Mtrr* gene) using published ChIP-seq data sets[4] in wildtype embryonic stem cells (ESCs) and trophoblast stem cells (TSCs). Dark grey rectangles indicate enriched peaks for each histone mark. Red rectangle and light grey shading indicate *Cwc27* differentially methylated region (DMR) identified in sperm of *Mtrr*$^{+/gt}$ males. Green rectangles indicate enhancer regions. Partial schematic of gene encoding for *Cwc27* is shown.

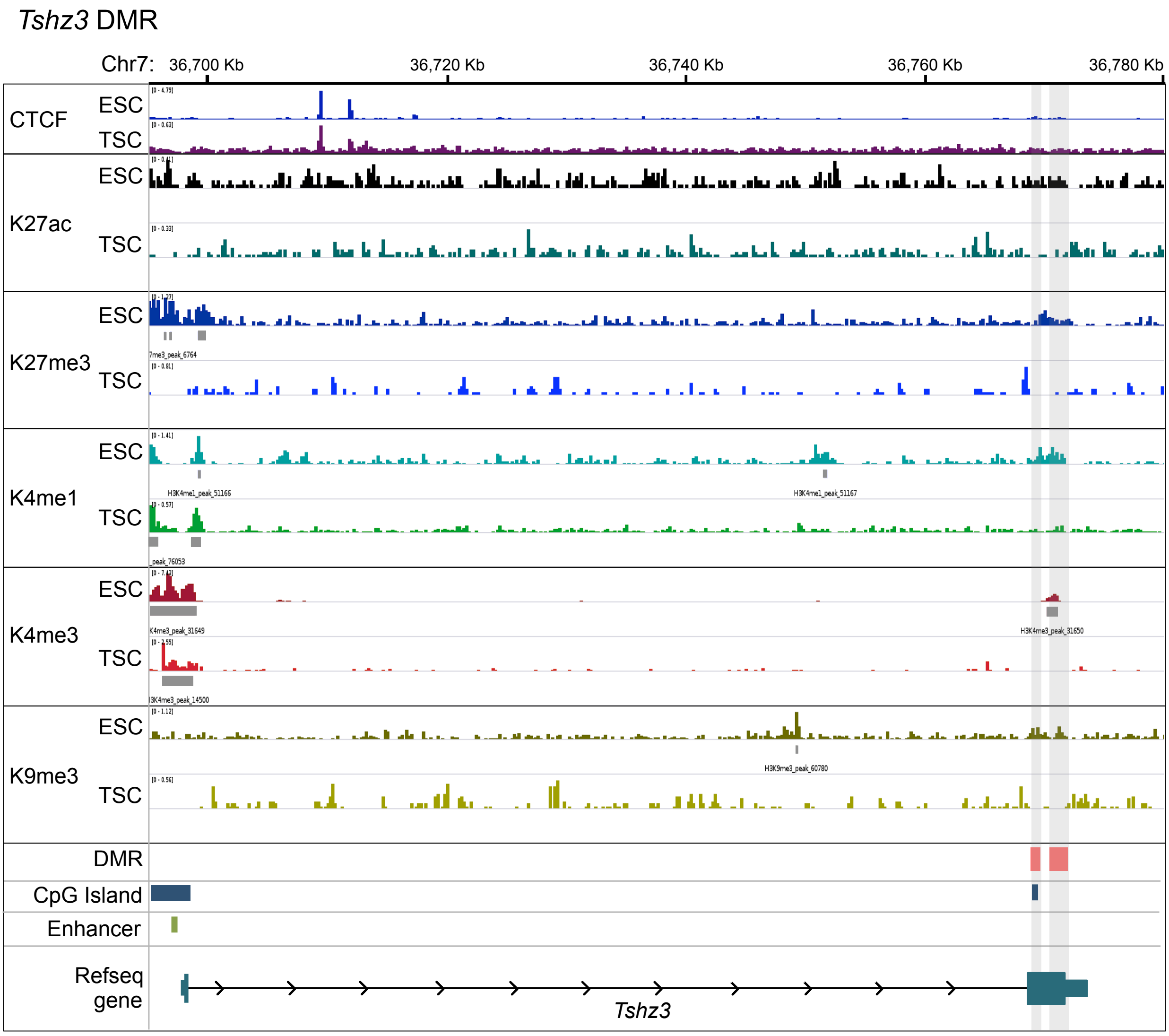

Supplementary Figure 10 **Epigenetic signature of the intragenic *Tshz3* DMR in wildtype ESCs and TSCs.** Enrichment of DNA binding proteins (CCCTC-binding factor, CTCF) and histone modifications (H3K27ac, H3K27me3, H3K4me1, H3K4me3, H3K9me3) in the *Tshz3* (teashirt zinc finger family member 3) locus on chromosome (Chr) 7 using published ChIP-seq data sets[4] in wildtype embryonic stem cells (ESCs) and trophoblast stem cells (TSCs). Dark grey rectangles indicate enriched peaks for each histone mark. Red rectangle and light grey shading indicate *Tshz3* differentially methylated region (DMR) identified in sperm of *Mtrr*$^{+/gt}$ males. Blue rectangles indicate CpG islands. Green rectangle indicates enhancer regions. Schematic of gene encoding for *Tshz3* is shown.

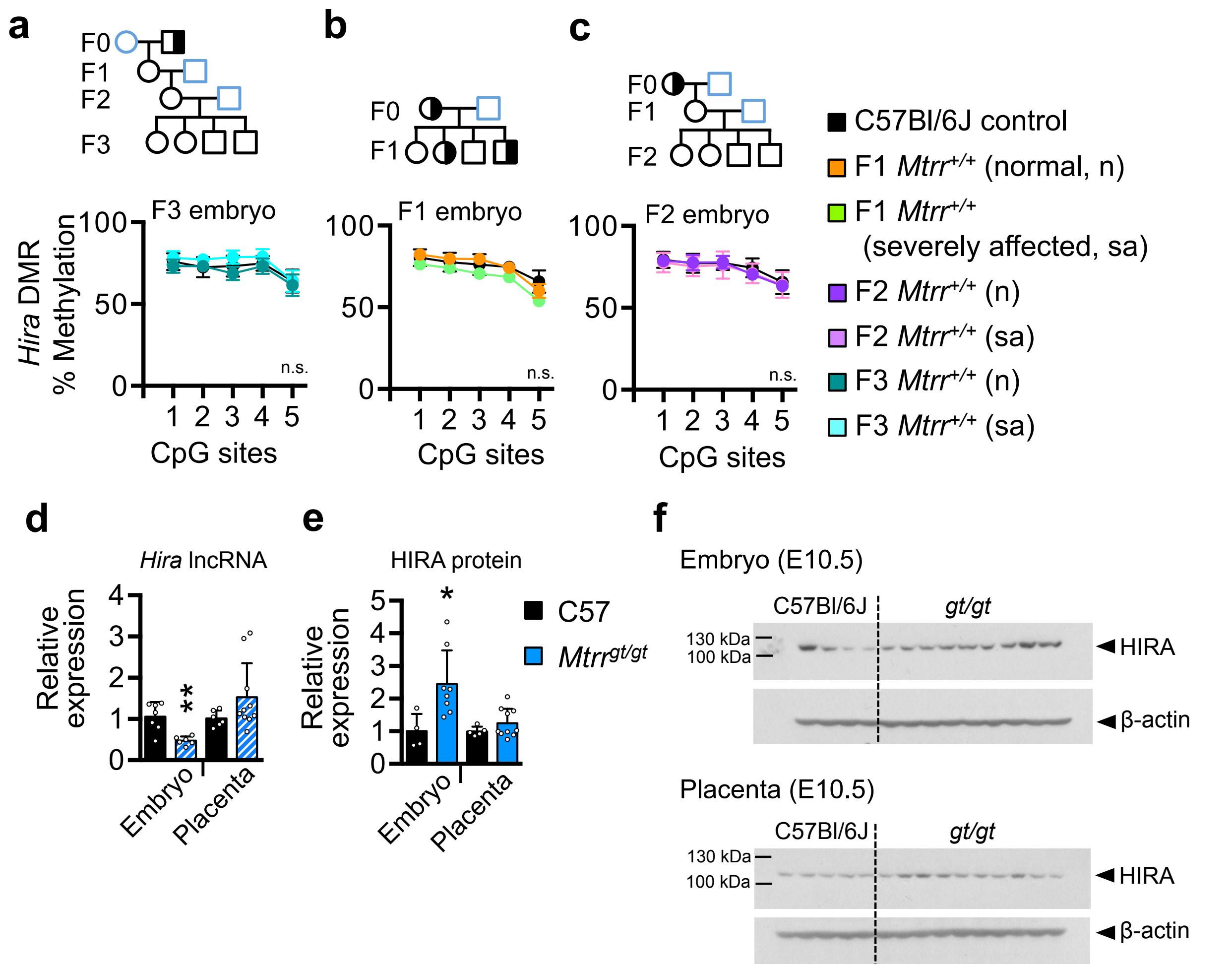

Supplementary Figure 11 **Analysis of *Hira* DMR methylation and HIRA protein expression. a-c** Bisulfite pyrosequencing analysis of average percentage methylation at individual CpG sites in the *Hira* (histone cell cycle regulator) DMR (see also Fig. 5) in C57Bl/6J embryos (N=6-8 embryos; black circles) at E10.5 compared to the following: **a** F3 wildtype (*Mtrr*$^{+/+}$) embryos at E10.5 derived from F0 *Mtrr*$^{+/gt}$ males (N=4 phenotypically normal (n) embryos teal circles; N=3 severely affected (sa) embryos, turquoise circles), **b** F1 wildtype (*Mtrr*$^{+/+}$) embryos at E10.5 derived from F0 *Mtrr*$^{+/gt}$ females (N=7 phenotypically normal (n) embryos, orange circles; N=3 severely affected (sa) embryos, green circles). **c** F2 wildtype (*Mtrr*$^{+/+}$) embryos at E10.5 derived from F0 *Mtrr*$^{+/gt}$ females (N=5 phenotypically normal (n) embryos, purple circles; N=4 severely affected (sa) embryos, pink circles). Data is presented as average methylation (mean ± standard deviation) per CpG site. Experiments were conducted in technical triplicates. Two-way ANOVA, with Sidak's multiple comparisons test, performed on mean methylation per CpG site. **d** RT-qPCR analysis of *Hira* lncRNA expression (striped bars, primer set 2) in embryos and placentas at E10.5 from C57Bl/6J

conceptuses (black bars, N=7 embryos, N=6 placentas) and *Mtrr*$^{gt/gt}$ conceptuses (blue bars, N=6 embryos, N=10 placentas). **e, f** Western blot analysis of HIRA protein in embryos and placentas at E10.5 from C57Bl/6J conceptuses (C57, black bars, N=4 embryos, N=5 placentas) and *Mtrr*$^{gt/gt}$ conceptuses (*gt/gt*; blue bars, N=8 embryos, N=10 placentas. Blots are shown in **f**. HIRA protein levels were normalised to β-actin loading control. Data in (**d**) and (**e**) were plotted as mean ± standard deviation and presented as relative expression to C57Bl/6J levels (normalized to 1). Experiments were conducted in technical duplicate (protein) or triplicate (RNA). Independent two-tailed t test, *p=0.0263, **p=0.0033. See also Fig. 6. Pedigree key: circles, females; squares, males; blue outline, C57Bl/6J control mouse line; black outline, *Mtrr*^{gt} mouse line; white fill, *Mtrr*$^{+/+}$; half white-half black fill, *Mtrr*$^{+/gt}$; black fill, *Mtrr*$^{gt/gt}$. See also Supplementary Fig 1 for details of breeding schemes. F0, parental generation; F1, first filial generation; F2, second filial generation; F3 third filial generation. Source data are provided as a Source Data file.

SUPPLEMENTARY REFERENCES

1. Watkins-Chow, D.E. & Pavan, W.J. Genomic copy number and expression variation within the C57BL/6J inbred mouse strain. *Genome Res* **18**, 60-6 (2008).
2. Hackett, J.A. *et al.* Germline DNA demethylation dynamics and imprint erasure through 5-hydroxymethylcytosine. *Science* **339**, 448-52 (2013).
3. Kobayashi, H. *et al.* Contribution of intragenic DNA methylation in mouse gametic DNA methylomes to establish oocyte-specific heritable marks. *PLoS Genet* **8**, e1002440 (2012).
4. Schoenfelder, S. *et al.* Divergent wiring of repressive and active chromatin interactions between mouse embryonic and trophoblast lineages. *Nat Commun* **9**, 4189 (2018).
5. Jung, Y.H. *et al.* Chromatin States in Mouse Sperm Correlate with Embryonic and Adult Regulatory Landscapes. *Cell Rep* **18**, 1366-1382 (2017).
6. Smith, Z.D. *et al.* Epigenetic restriction of extraembryonic lineages mirrors the somatic transition to cancer. *Nature* **549**, 543-547 (2017).
7. Gillich, A. *et al.* Epiblast stem cell-based system reveals reprogramming synergy of germline factors. *Cell Stem Cell* **10**, 425-39 (2012).
8. Rameix-Welti, M.A. *et al.* Visualizing the replication of respiratory syncytial virus in cells and in living mice. *Nat Commun* **5**, 5104 (2014).
9. Kim, S. *et al.* PRMT5 protects genomic integrity during global DNA demethylation in primordial germ cells and preimplantation embryos. *Mol Cell* **56**, 564-79 (2014).
10. Tuorto, F. *et al.* RNA cytosine methylation by Dnmt2 and NSun2 promotes tRNA stability and protein synthesis. *Nat Struct Mol Biol* **19**, 900-5 (2012).
11. Padmanabhan, N. *et al.* Mutation in folate metabolism causes epigenetic instability and transgenerational effects on development. *Cell* **155**, 81-93 (2013).
12. Tunster, S.J. Genetic sex determination of mice by simplex PCR. *Biol Sex Differ* **8**, 31 (2017).
