# Supplementary material for: Defective folate metabolism causes germline epigenetic instability and distinguishes Hira as a phenotype inheritance biomarker
Source: Nat Commun. 2021 Jun 17;12:3714. doi: 10.1038/s41467-021-24036-5 (PMC8211854; doi:10.1038/s41467-021-24036-5)
Supplement: Supplementary file 3 — Reporting Summary [file 41467_2021_24036_MOESM3_ESM.pdf]

nature research

Corresponding author(s): Erica D Watson

Last updated by author(s): Apr 19, 2021

Reporting Summary

Nature Research wishes to improve the reproducibility of the work that we publish. This form provides structure for consistency and transparency in reporting. For further information on Nature Research policies, see our Editorial Policies and the Editorial Policy Checklist.

Statistics

For all statistical analyses, confirm that the following items are present in the figure legend, table legend, main text, or Methods section.

n/a	Confirmed	
☐	☒	The exact sample size (n) for each experimental group/condition, given as a discrete number and unit of measurement
☐	☒	A statement on whether measurements were taken from distinct samples or whether the same sample was measured repeatedly
☐	☒	The statistical test(s) used AND whether they are one- or two-sided<br>*Only common tests should be described solely by name; describe more complex techniques in the Methods section.*
☒	☐	A description of all covariates tested
☐	☒	A description of any assumptions or corrections, such as tests of normality and adjustment for multiple comparisons
☐	☒	A full description of the statistical parameters including central tendency (e.g. means) or other basic estimates (e.g. regression coefficient) AND variation (e.g. standard deviation) or associated estimates of uncertainty (e.g. confidence intervals)
☐	☒	For null hypothesis testing, the test statistic (e.g. F, t, r) with confidence intervals, effect sizes, degrees of freedom and P value noted<br>*Give P values as exact values whenever suitable.*
☒	☐	For Bayesian analysis, information on the choice of priors and Markov chain Monte Carlo settings
☒	☐	For hierarchical and complex designs, identification of the appropriate level for tests and full reporting of outcomes
☒	☐	Estimates of effect sizes (e.g. Cohen's d, Pearson's r), indicating how they were calculated

Our web collection on statistics for biologists contains articles on many of the points above.

Software and code

Policy information about availability of computer code

Data collection	AxioVision 4.7.2 software (Carl Zeiss) imaging software program was used to obtain images of conceptuses.
Data analysis	The code used in this study are available https://github.com/CTR-BFX/Blake_Watson.<br>Bioinformatics software used:<br>FastQC version 0.11.5<br>TrimGalore version 0.6.4<br>bedtools2 version 2.27.0<br>bcftools version 1.3.1<br>bowtie2 version 2.3.4<br>Picard Tools version 2.9.0<br>multiqc version 1.4<br>bwa version 0.7.15-r1144-dirty<br>GenomeAnalysisTK version 3.7<br>R version 3.4.2<br>seqkit version 0.8.0<br>snpEff version 4.3t<br>vcftools version 0.1.15<br>Manta version 0.29.6<br>MEDIPS R package version 1.40.0<br>Repeatmasker version 4.1.1<br>Liftover version 20190415

deeptools 2.3.1

The following database was used:
FANTOM5 (https://fantom.gsc.riken.jp/5/)

AxioVision 4.7.2 (Carl Zeiss) imaging software program was used to obtain crown-rump lengths.
Pyrosequencing primers were designed using PyroMark Assay Design Software 2.0 (Qiagen).
Analysis of CpG methylation status was performed using Pyro Q-CpG software version 1.0.9 (Biotage).
ImageJ (64-bit) software version 1.48 (NIH, USA) was used to analyse western blot data.
Graphs were generated and statistical analyses were performed using GraphPad Prism 7 software.

For manuscripts utilizing custom algorithms or software that are central to the research but not yet described in published literature, software must be made available to editors and reviewers. We strongly encourage code deposition in a community repository (e.g. GitHub). See the Nature Research guidelines for submitting code & software for further information.

Data

Policy information about availability of data

All manuscripts must include a data availability statement. This statement should provide the following information, where applicable:

- Accession codes, unique identifiers, or web links for publicly available datasets
- A list of figures that have associated raw data
- A description of any restrictions on data availability

The whole genome sequencing data set has been deposited in ArrayExpress database at EMBL-EBI under accession number E-MTAB-8513 (https://www.ebi.ac.uk/arrayexpress/experiments/E-MTAB-8513) and the MeDIP-Seq data accession number is E-MTAB-8533 (https://www.ebi.ac.uk/arrayexpress/experiments/E-MTAB-8533). The published Hi-C data set accession number is E-MTAB-6585 (https://www.ebi.ac.uk/arrayexpress/experiments/E-MTAB-6585/ PMID:30305613). The source and accession numbers of processed ChIP-seq and ATACseq wig/bigwig files are listed in Supplementary Table 10 and accessible on GitHub (https://github.com/CTR-BFX/Blake_Watson). The Source Data for Fig 1b, 1d, 1e, 2a, 2d, 3a-j, 4a-f, 5c, 6a-f, Supplementary figures 4a-b, 5b-c, 8a-m, 11a-f are provided as a Source Data file. All relevant data are available from the corresponding author upon reasonable request.

Field-specific reporting

Please select the one below that is the best fit for your research. If you are not sure, read the appropriate sections before making your selection.

☒ Life sciences ☐ Behavioural & social sciences ☐ Ecological, evolutionary & environmental sciences

For a reference copy of the document with all sections, see nature.com/documents/nr-reporting-summary-flat.pdf

Life sciences study design

All studies must disclose on these points even when the disclosure is negative.

Sample size	N values were chosen based on accepted practices and routinely employed sample sizes for respective experiments including in our previous publications (PMID:24074862, PMID:25011554). N values are listed in the figure legends and where not possible, in Supplementary table 7.
Data exclusions	No data was excluded.
Replication	Experiments were repeated where possible. RT-qPCR and pyrosequencing results were performed using technical duplicates or triplicates. Results were confirmed by independent biological samples as indicated in the figure legends and Supplementary table 7.
Randomization	Experimental groups were determined by pedigree, genotype, sex, and phenotype criteria (where applicable). Individuals from different parents were selected where possible. Conceptuses were chosen at random within these narrow criteria.
Blinding	The investigators were not blinded to group allocation during experiments and outcome assessment due to the experimental design in which specific pedigree, genotype, and phenotype groups were purposefully selected for analysis. WGS and MeDIP-seq was performed blind to genotype of experimental groups.

Reporting for specific materials, systems and methods

We require information from authors about some types of materials, experimental systems and methods used in many studies. Here, indicate whether each material, system or method listed is relevant to your study. If you are not sure if a list item applies to your research, read the appropriate section before selecting a response.

Materials & experimental systems

n/a	Involved in the study	
☐	☒	Antibodies
☒	☐	Eukaryotic cell lines
☒	☐	Palaeontology and archaeology
☐	☒	Animals and other organisms
☒	☐	Human research participants
☒	☐	Clinical data
☒	☐	Dual use research of concern

Methods

n/a	Involved in the study	
☒	☐	ChIP-seq
☒	☐	Flow cytometry
☒	☐	MRI-based neuroimaging

Antibodies

Antibodies used	OptimAB mouse anti-human 5-methylcytosine (clone 33D3), Eurogentec Ltd., Cat No. BI-MECY-0100, RRID:AB_2616058<br>Rabbit anti-human HIRA (clone D2A5E), Cell Signaling Technology, Cat No. 13307, RRID:AB_2798177<br>Mouse anti-human beta-actin (clone AC-74), Sigma-Aldrich, Cat No. A2228, RRID:AB_476697<br>Donkey anti-rabbit IgG conjugated to horse radish peroxidase (HRP; clonality unknown), GE Healthcare, Cat. No. NA934, RRID:AB_772206<br>Sheep anti-mouse IgG conjugated to HRP (clonality unknown), GE Healthcare, Cat. No. NA931, RRID:AB_772210
Validation	anti-5mC - PMID: 23034951<br>anti-HIRA: https://www.cellsignal.jp/datasheet.jsp?productId=13307&images=1&protocol=0<br>anti-beta-actin: this antibody has been extensively validated. Studies can be found at the following link: https://antibodyregistry.org/search.php?q=AB_476697

Animals and other organisms

Policy information about studies involving animals; ARRIVE guidelines recommended for reporting animal research

Laboratory animals	Mtrr Gt(XG334)Byg (MGI:3526159) mice were from Erica Watson's laboratory, backcrossed into a C57Bl/6J background, and were described previously (PMID: 17369066; PMID: 24074862). C57Bl/6J mice were originally purchased from The Jackson laboratories (www.jaxmice.jax.org) and 129P2Ola/Hsd mice were purchased from Envigo (previously Harlan Laboratories [www.envigo.com]). All mice were bred and maintained in house at the University of Cambridge animal facility under a temperature- and humidity-controlled environment with a 12 hr light/dark cycles. Mice were provided with food and water ad libitum. Details of breeding pedigrees are listed in the manuscript and specifically in Supplementary figure 1. Male and female mice were assigned to experimental groups according to genotype and phenotype. Sperm and liver samples were collected from 16-20 week-old males. Pregnant females and their mates were younger than six months of age, and all embryos and placentas were collected at embryonic day (E) 10.5. A mix of male and female conceptuses were assessed as no phenotypic or molecular sexual dimorphism is evident in the Mtrr gt mouse line at E10.5 (PMID: 24074862; PMID: 29492317).
Wild animals	No wild animals were used.
Field-collected samples	No field-collected samples were used.
Ethics oversight	This research was regulated under the Animals (Scientific Procedures) Act 1986 Amendment Regulations 2012 following ethical review by the University of Cambridge Animal Welfare and Ethical Review Body.

Note that full information on the approval of the study protocol must also be provided in the manuscript.
